# The Vital@Work Study. The systematic development of a lifestyle intervention to improve older workers' vitality and the design of a randomised controlled trial evaluating this intervention

**DOI:** 10.1186/1471-2458-9-408

**Published:** 2009-11-10

**Authors:** Jorien E Strijk, Karin I Proper, Allard J van der Beek, Willem van Mechelen

**Affiliations:** 1 Department of Public and Occupational Health and the EMGO Institute for Health and Care Research, VU University Medical Center, van der Boechorststraat 7, 1081 BT Amsterdam, The Netherlands; 2 Body@Work, Research Center Physical Activity, Work and Health, TNO-VUmc, Amsterdam, The Netherlands

## Abstract

**Background:**

A major contributor of early exit from work is a decline in health with increasing age. As healthy lifestyle choices contribute to better health outcomes, an intervention aimed at an improved lifestyle is considered a potentially effective tool to keep older workers healthy and vital, and thereby to prolong labour participation.

**Methods:**

Using the Intervention Mapping (IM) protocol, a lifestyle intervention was developed based on information obtained from 1) literature, 2) a short lifestyle questionnaire aimed at indentifying the lifestyle behaviours among the target group, and 3) focusgroup (FG) interviews among 36 older workers (aged 45^+ ^years) aimed at identifying: a) key determinants of lifestyle behaviour, b) a definition of vitality, and c) ideas about how vitality can be improved by lifestyle.

The main lifestyle problems identified were: insufficient levels of physical activity and insufficient intake of fruit and vegetables. Using information from both literature and FG interviews, vitality consists of a mental and a physical component. The interviewees suggested to improve the mental component of vitality by means of relaxation exercises (e.g. yoga); physical vitality could be improved by aerobic endurance exercise and strength training.

The lifestyle intervention (6 months) consists of three visits to a Personal Vitality Coach (PVC) combined with a Vitality Exercise Programme (VEP). The VEP consists of: 1) once a week a guided yoga group session aimed at relaxation exercises, 2) once a week a guided aerobic workout group session aimed at improving aerobic fitness and increasing muscle strength, and 3) older workers will be asked to perform once a week for at least 45 minutes vigorous physical activity without face-to-face instructions (e.g. fitness). Moreover, free fruit will be offered at the group sessions of the VEP. The lifestyle intervention will be evaluated in a RCT among older workers of two major academic hospitals in the Netherlands. At baseline, after 6 and 12 months, measurements (primary: lifestyle and vitality, and secondary: work-engagement and productivity) will take place.

**Discussion:**

The lifestyle programme is developed specifically tailored to the needs of the older workers and which is aimed at improving their vitality.

**Trial registration:**

NTR1240

## Background

One of the most notable current and near future changes in the working population is its ageing. The baby boom cohort will start retiring in the coming decade. At the same time, fewer young people will enter the labour market due to lower birth rates in the past few decades [1]. These demographic changes will cause a shift in the ratio of workers-retirees, leading to a relative shortage of active labour force. As a consequence, a shrinking number of economically active people (i.e. workers) will have to pay for the national pensions of an increasing number of retired persons. In addition, many older people leave their job earlier than the official age of retirement [2]. To overcome these consequences, there is a need to find means for prolonging healthy labour participation of older workers. One of the major contributors to early exit from work is a decline in health [3-6]. Therefore, interventions aimed at the promotion of health may increase labour participation of older workers.

The World Health Organisation (WHO) has described health as "a state of complete physical, mental, and social well-being and not merely the absence of disease or infirmity" (WHO 1948). Despite lack of a sound documentation, it is assumed that vitality is closely related to this definition of health. Since it is no longer under debate that healthy lifestyle choices contribute to better health outcomes, an intervention aimed at improving lifestyle is considered as a potentially effective tool to keep older workers healthy and vital and thereby to contribute to prolonged employability. To date, no such lifestyle intervention exists.

The aim of the Vital@Work study is twofold, namely 1) to develop a lifestyle intervention to keep older workers vital, and 2) to scientifically evaluate the developed intervention. Consequently, this paper first describes the development and implementation of the lifestyle intervention, following the structure given by the Intervention Mapping (IM) protocol. Second, the design of the (cost-) effectiveness study evaluating the lifestyle intervention is described.

## Methods

### The systematic development of the lifestyle intervention

**Figure 1:**
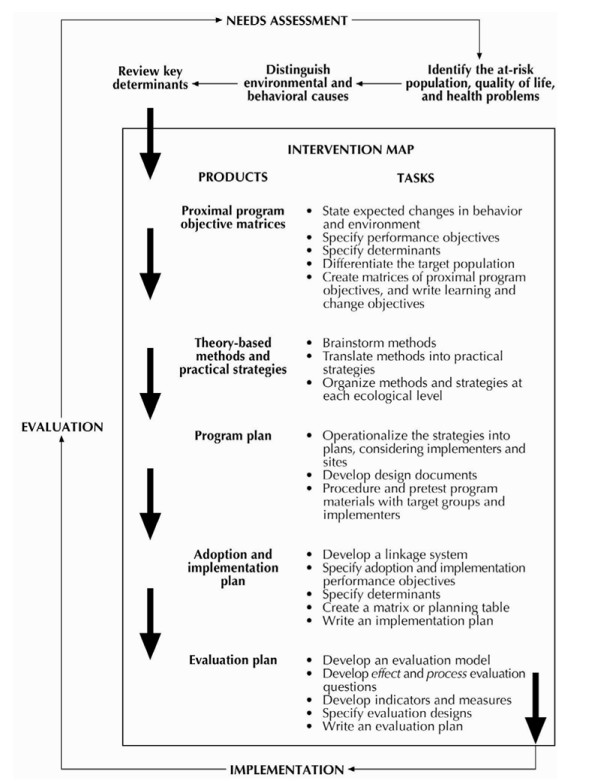
**The Intervention Mapping Process**. Source: Bartholomew *et al*. (2001).

The lifestyle intervention was developed using the Intervention Mapping (IM) protocol, which is a stepwise process for theory- and evidence-based development of a health promotion intervention (figure 1) [7]. IM is not a theoretical or conceptual framework, but rather a description of a logical planning process. It consists of six steps: 1) the needs assessment; 2) the definition of programme objectives, 3) the selection of adequate theories and methods, 4) the design of the intervention program, 5) the development of a plan for the adoption and implementation, and 6) the development of a evaluation plan.

#### Step 1 - Needs assessment

The lifestyle behaviours and associated health and vitality problems among older workers, were assessed by a thorough literature search as well as by information obtained from a short lifestyle questionnaire and focus group interviews. The needs assessment further gained insight into the definition of vitality and its association with lifestyle. The purpose of step 1 was to formulate the programme objectives of the lifestyle intervention.

Literature was searched to obtain information about older workers' lifestyle, the definition of vitality, and the association between lifestyle and vitality. A search for relevant scientific literature was conducted using MEDLINE. The key words used involved a combination of concepts regarding the study population (e.g. older workers, ageing workers, aging workers, older working population, middle aged), intervention (e.g. randomised controlled trial, evaluation, effect) and regarding the outcome measurements (e.g. vital, vitality, lifestyle, dietary habits, fruit intake, physical activity, relaxation). To obtain information about older workers' lifestyle, a random sample of 72 older workers (aged 45+) of a major Dutch academic hospital received, together with the invitation for the focusgroup interviews, a short lifestyle questionnaire. This questionnaire contained questions about physical activity, diet, and smoking. Participants were asked to return the questionnaire within a week. Completed questionnaires (n = 35) were analysed to gain insight into the main problems concerning the three lifestyle behaviours. Based on the lifestyle outcomes, semi-structured questions were formulated, specifically for the target group, to be discussed in the focus group interviews.

Older workers who completed the short lifestyle questionnaire received a confirmation for participation in a focusgroup interview. The aims of the focus group interviews were:

1) Identifying key determinants of the lifestyle behaviours,

2) Identifying ideas about the definition of vitality,

3) Identifying ideas about how vitality can be improved by a lifestyle intervention

Per aim, participants were asked to individually write down keywords on post-its (one keyword per post-it), without discussing their answers with other participants. Next, post-its were collected by the discussion leader. Underlying ideas of given answers (i.e. keywords) were further discussed during the focus group interview. In total, five focus group interviews with older workers (n = 32) were carried out.

#### Step 2 - Performance objectives

During this step of the IM process, the intervention was further tailored to the specific needs of the older workers. In addition, performance objectives were specified, which were based on the programme objectives identified during the needs assessment. Performance objectives are the effects of the intervention on the older workers in terms of what should be learned or which specific behaviour should be changed.

#### Step 3 - Theory-based methods and practical strategies

In step 3, theory-based methods and practical strategies that are likely to create the expected changes in the determinants were indentified. A method is a theory-based technique to influence change in determinants of behaviour or environmental conditions, whereas practical strategies are defined as techniques for the application of the theoretical methods. Methods and strategies were chosen based on the key determinants for the performance objectives, as selected in the needs assessment.

#### Step 4 - Design of the intervention

The next step of the IM process involved a description of the scope and sequence of the components of the lifestyle intervention. During the design of the intervention, primary aims of the lifestyle programme were formulated. Furthermore, methods and strategies selected in the previous step were translated into programme materials. Also, the intervention protocols were completed.

#### Step 5 - Adoption and implementation plan

The focus of step 5 is program adoption and the development of a plan for the implementation of the lifestyle programme. Facilitating factors and barriers regarding the adopting and implementation of the lifestyle programme were identified during the focus group interviews using semi-structured questions.

#### Step 6 - Evaluation plan

To assess the (cost)-effectiveness of the Vital@Work lifestyle programme, an evaluation plan was developed. The last step of the IM process describes the study design, study population, randomisation procedure, sample size, outcome measures, and statistical analysis.

### The design of the Vital@Work study: results from the IM process

#### Step 1 - Needs assessment

**Figure 2:**
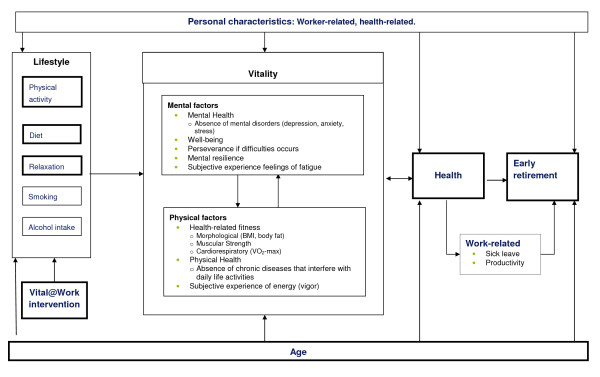
**The Vital@Work model, describing the Vital@Work intervention, aimed at improving lifestyle (physical activity, diet and relaxation), as a potentially effective tool to keep older workers vital and healthy and thereby contributing to prolonged employability**.

The starting point of the needs assessment was the current and near future ageing of the working population. Ageing is characterised by an increased prevalence of various chronic diseases such as CVD and cancer, but also by musculoskeletal disorders. As presented in the Vital@Work model (figure 2), a decline in health is one of the major contributors of early exit from work [3-6]. One concept that is assumed to be closely related and therefore might influence health is vitality. As healthy lifestyle choices contribute to better health outcomes, lifestyle is considered to influence older workers' vitality.

##### Vitality

Despite lack of an unambiguous definition of vitality in the scientific literature, it can be concluded that vitality is a comprehensive and complex concept, which is commonly used as a blanket term consisting of several factors. For example, McNair et al. (1971) stated that vitality is "a mood of vigorousness and high energy" [8]. Ware & Sherbourne (1992) linked the concept of vitality to "one's energy level and fatigue" and indicated that vitality reflects general physical and mental well-being [9]. Ryan & Frederick (1997) described vitality as "a dynamic aspect of well-being, marked by subjective experience of energy and aliveness" [10]. In the specific field of occupational health, vitality has been described by Schaufeli & Bakker (2003) as one of the three dimensions of work engagement [11]. They characterised vitality by "high levels of energy, feeling strong and fit, mental resilience while working, the willingness to invest effort in one's work, and persistence even in the face of difficulties" [11,12]. Considering the definitions mentioned above, it becomes clear that vitality consists of a mental as well as a physical component (figure 1). As to the mental component, vitality reflects well-being, less feelings of fatigue, mental resilience, and perseverance. With respect to the physical component, vitality is characterised by high energy levels and feeling "strong and fit". The physical component of vitality can, in fact, also be considered as part of the health-related fitness construct as described by Bouchard et al. (1994) consisting of aerobic fitness (VO2-max), muscular strength, flexibility, and body composition (BMI, body fat) [13,14].

Similar findings according the definition of vitality, were found during the focus group interviews. The interviewees confirmed that vitality in their view consists of a mental and a physical component. According to the interviewees, the mental factors of vitality are characterised by mental health (absences of mental disorders, such as depression), well-being (e.g. being happy with yourself, being positive, being motivated), perseverance if difficulties occurs, and fatigue. The physical factors of vitality were, according to the interviewees, characterised by physical health (i.e. the absence of chronic diseases that interfere with daily life activities), feelings of energy, and having high levels of aerobic fitness.

##### Lifestyle & vitality

Lifestyle consists of several factors, including physical activity, food intake, relaxation, smoking, and alcohol intake. The effects of sufficient levels of physical activity on health are beyond doubt. Those with a physically active lifestyle are at decreased risk for various chronic diseases, such as cardiovascular disease (CVD), some types of cancer, type 2 diabetes, and obesity [13,15-18]. In addition, physical activity favourably affects mental health, well-being, and feelings of fatigue [19-22]. It has been shown that people who lead an active lifestyle for several years are at reduced risk for suffering symptoms of depression [22]. As described by Bouchard and colleagues (11), the positive effects of physical activity on health can be explained either through a direct relationship or an indirect one, namely through improved levels of health-related fitness (i.e. BMI, VO_2_-max, muscle strength). As to the indirect relationship, the physical activity level should be of at least vigorous intensity, and of a certain frequency and duration.

Next to physical activity, an unhealthy diet rich in saturated fat intake and a low intake in fruit and vegetables is associated with several chronic diseases, such as CVD and cancer [21,23,24]. In particular, fruit and vegetable consumption have shown to be associated with lower levels of blood pressure and a lower risk of hypertension [25].

Another lifestyle factor that has shown to be associated with a reduction in blood pressure is relaxation [26,27]. There are also indications that relaxation leads to a reduction in coronary heart disease [26,27]. Moreover, a six-month lasting relaxation exercise program (yoga) has proven to yield favourable effects on well-being, experienced energy levels, quality of life, perceived stress, anxiety, and fatigue [28].

As to smoking, many epidemiological studies have shown the negative health consequences, namely elevated risks for all-cause mortality, CVD, cancer, and respiratory diseases, such as COPD [29-34].

Finally, the negative health outcomes for high alcohol consumption are all-cause mortality, and an increased risk for various diseases, such as CVD, stroke, diabetes, and liver disease [35-38].

##### Problems concerning lifestyle

More than 60 percent of all Dutch adults do not achieve the recommended amount of physical activity (i.e. 5 days a week 30 minutes of moderate intensity physical activity, and 3 days a week 20 minutes of vigorous intensity physical activity) [18,39]. In fact, 25 percent of all adults are not active at all [18].

Regarding fruit and vegetables, Dutch authorities have recommended intake levels of 200 g of vegetables and two pieces of fruit per day [40,41]. Similar amounts are recommended in other Western countries [42]. However, many consumers do not meet these recommendations [43]. Estimated current smokers among adults are in the Netherlands is 29.6 percent, and in the US 19.6 percent [44,45]. In contrast to the general population, no detailed information about older workers' unhealthy lifestyle could be indentified in the scientific literature. However, based on the lifestyle questionnaire pilot data (n = 35) two main problems concerning older workers' lifestyle were identified: the older workers' had insufficient levels of physical activity and they did not eat the daily recommended amount of fruit.

##### Improving vitality by a lifestyle intervention

To gain insight into the thoughts of older workers about how vitality can be improved by a lifestyle intervention, a distinction was made during the focus group interviews for an intervention focussed at the mental and at the physical factors of vitality. According to the interviewees, mental factors of vitality can be improved by activities that are aimed at relaxation. Most frequently mentioned ideas for improving mental vitality were participation in lessons of yoga, Tai Chi, and meditation. Further, interviewees reported the wish to learn performing those relaxation exercises at the workplace. The interviewees indicated that the physical factors of vitality can be improved by physical activities aimed at improving aerobic fitness. Interventions focussed at improving physical vitality mentioned frequently included fitness (both fitness exercise classes and individually), walking, and cycling. Based on scientific literature and expertise, physical activity levels should be of at least vigorous intensity to obtain improved aerobic fitness levels [13,46]. Since muscle strengths is, besides improving aerobic fitness levels, also associated with improved health outcomes such as reduced risk of cardiac vascular diseases (CVD) and muscoskeletal diseases, there should also be a focus on improving muscle strengths [47,48].

Also, the content and way of delivering of the intervention were specified by the older workers during the focus group interviews. Older workers mentioned their preferences about the timing: training sessions should be offered around working hours (lunchtime and after working hours), two times a week, and should last about 30-40 minutes. They further preferred guided small group lessons for social interaction, as well as for coaching about how to perform exercises without getting injured. Besides a group-based approach, an individual approach was desired for providing physical activity and dietary advice. Another condition that was mentioned was flexibility: not having obligations towards others, flexible times, and having the possibility to chose different kinds of physical activities.

Ideas mentioned by the older workers about how vitality can be improved by eating more fruit were offering free fruit and by taking a daily fruit break at a settled time.

##### Programme objectives

Based on the needs assessment, programme objectives were defined. These provide the foundation for the intervention by specifying who and what will change as a result of the lifestyle intervention.

The formulated programme objectives for the Vital@Work lifestyle intervention were:

1) Older workers will improve mental factors of vitality by relaxation exercises

2) Older workers will improve physical factors of vitality by vigorous intensity physical activity, i.e. by training

3) Older workers will improve their dietary habits by increasing daily intake of fruit

#### Step 2 - Performance objectives

The performance objectives formulated for the Vital@Work lifestyle intervention are presented in table 1.

**Table 1:** Performance objectives for the programme objectives formulated for the Vital@Work lifestyle intervention

**Program objective**	**Performance objective**
Older workers improve the mental factors of vitality by relaxation exercises	**1. **Older workers follow once a week a guided group lesson aimed at relaxation exercises
	
	**2. **Older workers monitor their weekly performed relaxation exercises
	
	**3. **Older workers are able to perform relaxation exercise on their own

Older workers improve the physical factors of vitality by vigorous intensity physical activity	**4. **Older workers follow once a week a guided group lesson aimed at vigorous intensity physical activity
	
	**5. **Older workers monitor their weekly vigorous physical activities
	
	**6.**Older workers identify barriers for not being vigorous physical active
	
	**7. **Older workers are able to identify solutions for the recognized barriers for not being vigorous physical active

Older workers improve their dietary habits by increasing daily intakes of fruit	**8. **Older workers increase their daily intakes of fruit
	
	**9. **Older workers monitor their weekly intake of fruit

##### Determinants of performance objectives

Based on the focus group interviews important and changeable determinants of the performance objectives were selected. The following determinants for physical activity (including both relaxation and vigorous intensity physical activity) were identified: intention, self-efficacy, attitude, habits, skills, awareness (of own physical activity level), and social support. The most important determinants of healthy dietary habits by eating daily a sufficient amount of fruit were: self-efficacy, attitude, habits, awareness, social norm, and intention.

#### Step 3 - Theory-based methods and practical strategy

Methods were selected in order to change the determinants selected for performance objectives formulated for the Vital@Work intervention. Selected methods were: guided practice, goal setting, environmental changes, decisional balance, (self) monitoring, and self-evaluation. Methods were translated into practical strategies in order to enable older workers to accomplish the performance objectives successfully. The methods and strategies for the first performance objective (older workers will improve mental factors of vitality by relaxation exercises) are presented in table 2. As an example, skills training (i.e. guided group sessions of yoga) were selected as a practical strategy to apply the method guided practice.

**Table 2:** Methods and strategies selected for improving older workers' mental factors of vitality by relaxation

**Determinant**	**Methods from Theory**	**Strategy**	**Tools/materials**
**Skills: Learning exercises to relax**	Guided practice	- Skills training	- Guided relaxation exercise sessions (Yoga)

**Self-Efficacy**	Goal Setting	- Formulation of implementation intentions - Planning coping responses - Individualized feedback	- Discussing worksheet to help planning goals (How? When?) during Personal Vitality Coach (PVC) visits - Discussion barriers/difficult situations, and possible solutions with PVC - Feedback on formulated goals (e.g. attended group sessions)
	
	Environmental changes	- Facilitation of healthy behaviour	- offering guided yoga session in the near environment of the workplace

**Attitude**	Decisional balance	- Knowledge	- Providing information (e.g. leaflet, PVC) of relaxation and its relation to health-related outcome (e.g. mental health, well-being)

**Habits**	Goal setting	- Formulation of implementation intentions	- Discussing worksheet to help planning goals (How? When?) during PVC visits

**Awareness**	Self-evaluation	- Monitoring own relaxation behaviour	- Relaxation diary

**Intention**	Goal Setting	- Formulation of implementation intentions	- Discussing worksheet to help planning goals (How? When?) during PVC visits

Methods and strategies for the second performance objective (older workers will improve physical factors of vitality by vigorous intensity physical activity) are presented in table 3. As an example, formulating implementation intentions (i.e. a worksheet to help planning personal goals by specifying how and when) and individualised feedback (i.e. discussion barriers and difficult situations with the Personal Vitality Coach) were selected as a practical strategies to apply the method goal setting.

**Table 3:** Methods and strategies for improving physical factors of vitality by increasing vigorous intensity physical activity

**Determinant**	**Methods from Theory**	**Strategy**	**Tools/materials**
**Skills: improving aerobic fitness**	Guided practice	- Skills training	- Guided workout sessions exercise

**Self-Efficacy**	Goal Setting	- Formulation of implementation intentions - Planning coping responses - Individualized feedback	- Discussing worksheet to help planning goals (How? When?) during Personal Vitality Coach (PVC) visits - Discussing barriers/difficult situations, and possible solutions with PVC - Feedback from PVC on formulated goals (e.g. attended group sessions)
	
	Environmental changes	- Facilitation of healthy behaviour	- Offering workout sessions in the near environment of the workplace

**Attitude**	Decisional balance	- Knowledge	- Providing information (e.g. leaflet, PVC) regarding physical activity

**Habits**	Goal setting	- Formulation of implementation intentions - Rewarding	- Discussing worksheet to help planning goals (How? When?) during PVC visits - Feedback from PVC on formulated goals

**Awareness**	Self-evaluation	- Monitoring of own physical activity	- Physical activity diary

**Intention**	Goal Setting	- Formulation of implementation intentions	- Discussing worksheet to help planning goals (How? When?) during PVC visits

Methods and strategies for the third performance objective (older workers will improve their dietary habits by increasing daily intake of fruit) are presented in table 4. As an example, monitoring own fruit intake (i.e. fruit diary) was selected for as a practical strategy to apply the method self-evaluation.

**Table 4:** Methods and strategies selected for improving dietary habits by increasing daily intakes of fruit

**Determinant**	**Methods from Theory**	**Strategy**	**Tools/materials**
**Self-Efficacy**	Goal Setting	- Formulation of implementation intentions - Individualized feedback - Planning coping responses	- Discussing worksheet to help planning goals (How? When?) during Personal Vitality Coach (PVC) visits - Discussion barriers/difficult situations, and possible solutions with PVC
	
	Environmental changes	- Facilitation of healthy behaviour	- Providing free fruit at the Vitality Exercise Programme (VEP)

**Attitude**	Decisional balance	- Knowledge	- Providing information (e.g. leaflet, coach) regarding the behaviour and connection between behaviour and (health-related) outcome

**Habits**	Goal setting	- Formulation of implementation intentions	- Discussing worksheet to help planning goals (How? When?) during PVC visits

**Awareness**	Self-evaluation	- Monitoring own fruit intake	- Fruit intake diary

**Intention**	Goal Setting	- Formulation of implementation intentions	- Discussing worksheet to help planning goals (How? When?) during PVC visits

#### Step 4 - design of the intervention

Based on the process of Intervention Mapping, the primary aims of the intervention are:

1) Improving older workers' mental factors of vitality by relaxation exercises;

2) Improving older workers' physical factors of vitality by vigorous intensity physical activity.

Additionally, the secondary aim of the intervention is to improve older workers' dietary habits by increasing intakes of fruit. The intervention will last 6 months and will consists of 1) three visits to a Personal Vitality Coach (PVC) combined with 2) the Vitality Exercise Programme (VEP), and 3) provided free fruit at the VEP.

##### Personal Vitality Coach (PVC) Visits

All participants will be invited to visit the PVC three times during 6 months. The first visit (30 minutes) will be at the start of the intervention, the follow-up visits (30 minutes) will be at 4-6 weeks and 10-12 weeks after the first PVC visit.

The coaching visits will be aimed at: 1) goal setting, 2) feedback on formulated goals (i.e. self-monitoring, self-evaluation), and 3) problem solving.

##### The Vitality Exercise Programme (VEP)

The VEP will be aimed at improving both mental and physical factors of vitality. Mental factors will be improved by yoga (relaxation exercises) and physical factors by a workout aimed at improving aerobic fitness and muscle strength. The VEP consist of:

1) a guided group session of yoga once a week,

2) a guided workout group session consisting of aerobic and resistance exercises once a week

3) aerobic exercising without direct face-to-face instruction. Attendance to this once a week additional session will be prescribed by the fitness instructor who guides the workout sessions, and by the PVC during the visits.

##### Yoga

Yoga will be guided by a qualified yoga instructor and will be provided once a week (in total 24 sessions during a intervention period of 6 months) in small group sessions (max. 16 persons) and consists of relaxation exercises. Each session will last 45 minutes and will start with relaxation and preparation postures for the hips, shoulders, neck, feed, and hands while focussing on breathing (5 minutes), followed by a series of standing postures, forward bending postures and twists, and light back bending postures (30 minutes). Each yoga session will be ended with an yoga exercise aimed at total relaxation, known as the 'Savasana Corpse Pose' and mediation (10 minutes).

##### Workout

The workout will be guided by a fitness instructor and will be provided once a week (in total 24 sessions during a intervention period of 6 months), and will be conducted in small group training sessions (max. 16 persons). Each session will last 45 minutes, and will start with a warming-up of 5 minutes followed by aerobic exercises (in total 2 × 10 minutes, 1 × 5 minutes = 25 minutes), resistance training (2 × 5 = 10 minutes), and a cooling-down (3 minutes).

Improvements in aerobic fitness (i.e. VO_2_-max) are directly related to the frequency, intensity, and duration of the activities. The intensity of the workout will meet the ACSM-guidelines, which recommends an intensity that equals 65-90% of the maximum heart rate (HR_max_) [47,49]. The resistance training will be progressive in nature and provides stimulus to all major muscle groups. The ACSM-guidelines recommend a repetition maximum (RM) of 10-15 repetitions of each exercise. The frequency of the resistance exercises was defined as 3 RM (3 × 10-15 repetitions). Each exercise will be performed with a load at which the repetition maximum can just be maintained [48].

##### Aerobic exercise without direct face-to-face instruction

Besides the yoga and workout sessions, older workers will be prescribed to perform once a week for at least 45 minutes of vigorous physical activity without face-to-face instructions (e.g. fitness, running, spinning). To achieve improvement in aerobic fitness, workers will be asked to exercise with an intensity similar to the guided workout sessions. As an illustration of this intensity, workers got the instruction to exercise with an intensity at which they become sweating and experienced increased respiration and heart beat.

##### Providing free fruit at the VEP

During the intervention period, free fruit will be provided at the guided yoga and workout group sessions of the VEP.

#### Step 5 - Adoption and implementation plan

Two main factors for adoption of the programme by the target population were identified, namely time and place. Therefore, the lifestyle programme was modified to fit within a common working day of the older workers by choosing adequate time schedules for the provided yoga and guided workout group sessions. Guided group sessions will be provided in two time blocks on all working days: 1) during lunchtime (3 sessions), and 2) after working hours (3 sessions). Furthermore, to increase the adoption of the lifestyle programme, the guided group sessions will be provided near the worksite (max. 5-10 minutes walk).

#### Step 6 - Evaluation plan

The (cost-) effectiveness of the lifestyle intervention developed in the preceding IM steps, will be evaluated in a randomised controlled trial (RCT) with two arms. During six months, employees in the intervention group will receive the lifestyle programme as described above. The control group will receive the same written information as the intervention group about a healthy lifestyle (physical activity, relaxation, fruit). Both groups will be measured at baseline, and after 6 and 12 months. The Medical Ethical Committee of both the VU University Medical Center (VUmc, Amsterdam, the Netherlands) and the Leiden University Medical Center (LUMC, Leiden, the Netherlands) approved the study protocol.

##### Study population

The study population consists of older workers (aged 45 years and over) from the VUmc and LUMC, working at least 16 hours a week.

##### Recruitment of the study population

First, all potential participants will receive an invitation letter at their home postal address together with an information package consisting of: 1) flyer with information about the study, 2) informed consent, 3) screener for exclusion criteria: i.e. the physical activity readiness questionnaire (PAR-Q), and 4) a stamped and addressed envelope for reply. Workers who are willing to participate in the study will be asked to return the signed informed consent together with the completed screener within a week. Subsequently, workers who meet the inclusion criteria and signed the informed consent will receive the baseline questionnaire together with an invitation for the UKK walk test at their home postal address. Workers who are not willing to participate in the study will be asked to give their reasons for not participating. Two weeks after the initial mailing, a postcard will be sent to thank respondents for returning their screener and informed consent. For non-respondents the back of this postcard will be used as a reminder and as a second opportunity to complete the screener and informed consent. To minimise non-response during the follow-up measurements, all participants will receive a pre-notice card a week before the measurement. Subsequently (i.e. within one week after the pre-notice card), all participants will receive the follow-up questionnaire and will receive an invitation for another UKK walk test.

##### Randomisation procedure

A computer-generated randomisation will be performed at individual level after baseline measurements are completed. Randomisation will be executed, after completing baseline measurements, by an independent researcher (i.e. research assistant) using Random Allocation Software (Version 1.0, May 2004, Isfahan University of Medical Sciences, Iran).

##### Power calculation

The sample size calculation will be based on differences between the intervention and control group with regard to changes in the mean vitality score, measured by the Utrecht Engagement Scale (UWES) (10). Based on a study among 10.000 Dutch and Belgian employees, the baseline mean vitality score (range 0-6) is assumed to be 3.99 (SD = 1.11) [12]. For the sample size needed, a difference in the vitality mean score of 10% between the intervention and control group after six months will be considered relevant. This means an average difference in the vitality mean score of 0.4 (SD 1.2) between both study groups. Assuming α = 0.05, power = 0.90, and two-sided tests, 189 participants per group will be needed. Taking into account a loss of follow-up of 15%, a sample size of 446 employees (223 employees in each group) needs to be included. Further, based on an initial response of 20% of the eligible workers (i.e. workers aged 45 and over), 2230 workers need to be approached and asked to participate in the study. In total, 3756 older workers of the two academic hospitals will be approached for participation, thereby ensuring sufficient statistical power, even in case of unexpectedly poor initial response and/or high loss to follow-up.

## Measurements

All measurements will be completed at baseline, after 6, and 12 months. The measurements will consist of 1) a questionnaire, containing questions concerning lifestyle, vitality, general health, work and the health-related fitness construct Body Mass Index (BMI) and waist circumference, combined with 2) the 2-km UKK walking test.

### Lifestyle

The level of physical activity will be assessed using the validated Short Questionnaire to AssesS Health enhancing physical activity (SQUASH) [50]. The SQUASH measures duration, frequency and intensity of four clusters of physical activity, i.e. commuting activities, household activities, activity at work, and leisure time activities. The SQUASH has been shown to be a fairly reliable and reasonably valid questionnaire [50].

In addition, physical activity will be measured objectively in a random sample of 200 participants of both the intervention (n = 100) and control group (n = 100). This subsample will receive during a period of one week (7 days) an accelerometer (GTM1 ActiGraph, ActiTrainer ActiGraph), which registers the actual physical activity during daytime. To minimize both the intrainstrument variability (the difference within a single accelerometer over multiple follow-up measurements) and interinstrument variability (differences between different accelerometers during a single measurement), workers receive every follow-up measurement the same accelerometers. The accelerometers will be worn during waking hours on the right hip and will be handed out after completing the questionnaire. Fruit intake will be assessed using an adapted version of the validated Short Fruit and Vegetable questionnaire [51]. The questionnaire consists of 10 questions: 6 about fruit consumption and 4 about consumption of vegetables. In this study, only the questions about fruit will be asked.

### Vitality

A 17-item questionnaire, called the Utrecht Engagement Scale (UWES), will be used to measure workers' work engagement. The UWES consists of three aspects: vitality (6 items), dedication (5 items), and absorption (6 items) [11,12,52].

Vitality will be assessed by the six items of the UWES that refer to high levels of energy and resilience, the willingness to invest effort, not being easily fatigued, and persistence in the face of difficulties.

### Health-related fitness

BMI will be calculated using self-reported weight and height. All participants will be asked to report their self-measured waist circumference in each questionnaire. For that aim, a measuring tape (range 0-135 cm) will be sent to all participants along with the questionnaires. Participants will have instructions on how to use the measuring tape and are asked to report their waist circumference to the nearest cm. Besides BMI and waist circumference, the aerobic fitness (VO_2_-max) of the older workers will be measured using the UKK 2-km walking test. The optimal way of measuring VO_2_-max is by a maximal exercise test (i.e. treadmill test). However, for regular use in many research and clinical setting, this may be impractical. For simplicity, suitability and social acceptability walking is an attractive exercise mode for the purposes of mass testing [53]. Therefore, in this study the UKK walk test will be used to predict VO_2_-max. The UKK walk test is a simple and safe test designed to measure the aerobic fitness of normally active men and women [54]. The UKK walk test is a fast 2-km walk supplemented with simple measurements (heart rate, BMI) and has shown to be a feasible and accurate method for predicting VO_2_-max in healthy 20-65 year old subjects [55]. A gender-specific prediction model including walking time, heart rate at the end of the walk, age and body mass index predicted 73-75% of the variance in VO_2_-max [53].

### General health

Information about whether or not suffering from any chronic diseases (e.g. cardiovascular diseases, respiratory diseases, muscoskeletal disorders, diabetes mellitus) will be obtained using a 1-item question about chronic diseases from the Dutch Working Conditions Survey [56]. Mental health will be measured using the 5-item scale of the RAND-36. Besides mental health, other health-related quality of life items will be measured: physical functioning (PH, 10 items), role limitations caused by physical problems (RP, 4 items), general health perceptions (GH, 5 items), and vitality (VT, 4 items) [57].

### Work-related outcomes

Sick leave will be determined using the World Health Organization Health and Work Performance Questionnaire (WHO-HPQ) measuring loss of productivity due to decreased performance while at work (presenteeism) and sick leave (absenteeism). This questionnaire has shown good concordance with employer records of work absenteeism and critical incidents [58,59]. For the specific purpose of the economic evaluation, participants will be asked to complete the WHO-HPQ once every three months. Besides loss of productivity, the need for recovery after a working day will be measured using the 11-item 'need for recovery scale' from the Dutch version of the Questionnaire on the Experience and Evaluation of Work (Dutch abbreviation VBBA) [60].

### Data analysis

#### Statistical analysis

The effectiveness of the lifestyle intervention will be analysed by means of a regression analysis (analysis of covariance) with the outcome measure at follow-up (6 months and 12 months) as the dependent variable and adjusting for the baseline levels of the outcome measure. Both crude and adjusted analyses will be performed. In the adjusted model, other potential confounders than covariates will be included, such as age, smoking and physical activity. Furthermore, effect modification, e.g. by gender, will be checked. All statistical analyses will be performed according to the intention-to-treat principle. For all analyses a two-tailed significance level of < 0.05 will be considered statistically significant. Linear and logistic (longitudinal) regression analyses will be performed with SPSS 15.0 (SPSS Inc. Chicago, Illinois, USA).

#### Economic evaluation

A cost-effectiveness analysis (CEA) will be performed from the company perspective. The time horizon will be 12 months, similar to the trial. The analysis will be performed according to the intention-to-treat principle. Missing data will be imputed using multiple imputation techniques. The primary outcome measurements in the CEA will be lifestyle (physical activity and daily fruit intake) and vitality (total score vitality scale UWES).

Two CEAs will be performed from the company perspective:

1) intervention costs together with savings as a result of reduced sick leave (absenteeism) and loss of productivity (presenteeism) will be compared to the obtained effects on the primary outcome measures lifestyle and vitality

2) intervention costs will be compared to the obtained benefits due to reduced sick leave and increased productivity.

#### Process evaluation

The process of the intervention will be evaluated according to the key process-relevant variables: recruitment, older workers' attitude towards the intervention, fidelity, reach, dose delivered, dose received, and implementation [61,62]. The recruitment of the older workers is described elsewhere in this article (see 'recruitment of the study population'). The other key process-relevant variables will be assessed in four ways. First, at post-test the attitude towards the intervention will be indentified among the older workers' in the intervention group by asking their opinion about: 1) the intervention (VEP and PVC) as a whole; 2) the coaches' competence, and 3) the effect of the intervention on their own subjective vitality. Second, by means of registration forms filled in by the PVCs during each coaching visit, attendance to the coaching protocol (fidelity) will be assessed. Third, older workers in the intervention group will be asked to keep up a physical activity and fruit diary (reach, dose received, fidelity). Finally, the VEP sessions which will be delivered by the providers will be defined at pre-test (see 'design of the intervention'). Additionally, the fitness instructors will be asked to register the presence of the older workers at the guided group sessions (reach, dose received, fidelity). On the process evaluation, quantitative analyses will be performed.

## Discussion

The aim of this article was to describe the development, and the design of the intended evaluation of a lifestyle programme aimed at improving vitality in older workers. Applying the IM protocol to develop the lifestyle programme required time and effort. However, it helped us to carefully consider each decision concerning the development of the lifestyle programme, and planning the implementation and evaluation of the lifestyle programme. Therefore, we perceived the use of the IM protocol as a useful tool that has guided us through the development of our lifestyle programme.

### Strengths and weaknesses

The rising prevalence of older workers in the near future has created a need for cost-effective interventions that can prolong healthy employability of older workers. Consequently, one of the main strengths of the Vital@Work study is that this is the first RCT that evaluates the effectiveness of a lifestyle intervention in order to improve both the mental en physical components of vitality. Another strength of the Vital@Work study is that as a result of applying the IM protocol, we developed a lifestyle programme taking into account both a theoretical framework and the perspectives of older workers on: 1) lifestyle, 2) vitality and, 3) how vitality can be improved by a lifestyle programme. We believe that this will lead to a better compliance to the lifestyle programme and therefore improve the likelihood of effectiveness of the lifestyle programme. However, limitations of this study can be mentioned. First, in this study only older workers from academic hospitals were involved in the focus group interviews. Therefore, it is possible that the IM process led to a lifestyle programme, which is only applicable to this specific target population. Second, it should also be noted that implementation of this lifestyle programme is likely to be more difficult in hospital settings where fitness facilities are not available in the direct environment.

### Comparison with other studies

This type of lifestyle programme has not been evaluated in the setting of academic hospitals and in this target group (workers aged 45 years and over) yet. In addition, the combination of vigorous intensity physical activity with relaxation exercises (yoga), has not been reported before. However, several studies have reported the effectiveness of worksite physical activity programs on physical health (e.g. aerobic fitness, BMI) [63-68]. In addition, only a small number of studies reported the effectiveness of worksite physical activity programs on mental health (e.g. well-being) [66]. The effectiveness of a lifestyle programme involving physical activity on vitality has not been evaluated before. Yoga appears to be effective in reducing stress and improving health status in adults [28,69]. However, there are no studies available reporting the effect of yoga or relaxation exercises in (older) workers.

## Conclusion

The development of the intervention according to the IM protocol resulted in a Vital@Work lifestyle programme specially tailored to the needs of older hospital workers. To determine the (cost-)effectiveness of the lifestyle programme, we will examine vitality, lifestyle behaviour (physical activity, relaxation, and fruit intake), work factors and aerobic fitness in a RCT. Results of the RCT will be available in 2011. If proven effective, both companies and society will benefit from a effective tool to keep older workers healthy and vital and thereby contribute to prolonged healthy employability.

## Competing interests

The authors declare that they have no competing interests.

## Authors' contributions

JES, KIP, AvdB, and WvM provided support in the design of the study and contributed intellectual input into the design of this paper. JES coordinated the process of application of the IM protocol to design the lifestyle programme. She performed data collection, analysed data and drafted the manuscript. KIP, AvdB, and WvM provided support during the development of the lifestyle programme. All authors contributed to the further writing of the manuscript. KIP, AvdB, and WvM obtained financial support. All authors have read and corrected draft versions of the manuscript and approved the final manuscript.

## Pre-publication history

The pre-publication history for this paper can be accessed here:


